# Randomized, Double-Blind, Reference-Controlled, Phase 2a Study Evaluating the Immunogenicity and Safety of OVX836, A Nucleoprotein-Based Influenza Vaccine

**DOI:** 10.3389/fimmu.2022.852904

**Published:** 2022-04-07

**Authors:** Isabel Leroux-Roels, Gwenn Waerlop, Jessika Tourneur, Fien De Boever, Catherine Maes, Jacques Bruhwyler, Delphine Guyon-Gellin, Philippe Moris, Judith Del Campo, Paul Willems, Geert Leroux-Roels, Alexandre Le Vert, Florence Nicolas

**Affiliations:** ^1^Center for Vaccinology (CEVAC), Ghent University and University Hospital, Ghent, Belgium; ^2^OSIVAX, Lyon, France

**Keywords:** influenza, universal vaccine, nucleoprotein, subunit, OVX836, Influvac Tetra, safety, immunogenicity

## Abstract

OVX836 is a recombinant protein-based vaccine targeting the highly conserved influenza nucleoprotein (NP), which aims to confer a broad-spectrum protection against influenza. In a Phase 1 study, OVX836, administered intramuscularly, has been found safe and immunogenic. The 90µg and 180µg dose levels were selected to be further evaluated in this randomized, monocenter, reference-controlled (Influvac Tetra™: quadrivalent seasonal influenza subunit vaccine), parallel group, double-blind, Phase 2a study in 300 healthy volunteers, aged 18-65 years, during the 2019/2020 flu season. Safety, influenza-like illness episodes (ILI; based on the Flu-PRO^®^ questionnaire) and immunogenicity were assessed up to 180 days post-vaccination. OVX836 was safe and presented a reactogenicity profile similar to Influvac Tetra. It induced a significant increase in terms of NP-specific interferon-gamma (IFNγ) spot forming cells (SFCs), NP-specific CD4+ T-cells (essentially polyfunctional cells) and anti-NP IgG responses. OVX836 was superior to Influvac Tetra for all immunological parameters related to NP, and the 180µg dose was significantly superior to the 90µg dose for SFCs and CD4+ T-cells expressing IFNγ. Both the CD4+ T-cell and the anti-NP IgG responses persisted up to Day 180. An efficacy signal was observed with OVX836 at 180µg through reduction of ILI episodes occurring during the flu season as of 14 days post-vaccination. In conclusion, these results encourage further clinical evaluation of OVX836 in order to confirm the signal of efficacy on ILIs and/or laboratory-confirmed influenza cases. NCT04192500 (https://clinicaltrials.gov/ct2/show/study/NCT04192500)

## Introduction

Influenza infection is a major cause of severe respiratory disease that affects all age groups, leading to 290,000 to 650,000 worldwide deaths annually (1).

Tetravalent influenza vaccines generating an antibody immune response mostly directed against the highly drifting hemagglutinin (HA) and neuraminidase (NA) envelope glycoproteins of the virus are the cornerstone of today’s influenza prevention (2). During the 2019/2020 influenza season, six influenza studies across Europe have indicated that vaccine effectiveness (VE) against any laboratory-confirmed influenza, across all ages, in primary care and hospital settings, ranged between 29% and 61%. VE estimates against influenza A/H3N2 were substantially lower (-58% to 57%) than against influenza A/H1N1 pdm09 (48% to 75%) and against influenza B (62 to 83%), while influenza A/H3N2 is generally associated with higher morbidity and mortality. Negative VE estimates for H3N2 could be due to immunological imprinting by first flu infection in childhood (3). There is therefore a need for vaccine efficacy improvement.

In addition to humoral immunity, cellular responses, in particular CD4 and CD8-mediated responses, very likely contribute to vaccine-induced protection (4–10). More specifically, in the Flu Watch Cohort Study, nucleoprotein (NP) specific T-cell levels above a threshold of 20 Spot Forming Cells [SFC]/million peripheral blood mononuclear cells [PBMC] at baseline measurement correlated with fewer cases of symptomatic, polymerase chain reaction (PCR)-positive influenza A, during both pandemic and seasonal influenza periods (11). As an internal protein, providing structural and functional support to the viral replication machinery (12), the influenza NP has a relatively low mutation rate throughout virus evolution (13, 14). The NP proteins of various virus isolates of the same influenza A subtype had over 90% amino acid homology (14, 15). Taken together, these data support the use of NP as a target to develop a broad-spectrum vaccine for the protection against influenza

OVX836 (OSIVAX, Lyon, France) is a universal vaccine candidate that demonstrated broad protection against multiple influenza A strains (16) and also cross-protection against B strains (unpublished data) in preclinical viral challenge models. OVX836 is a recombinant protein containing the full-length NP of the A/WSN/1933(H1N1) influenza virus and OVX313 (oligoDOM^®^), OSIVAX’s proprietary self-assembling nanoparticle technology (16, 17). This recombinant protein spontaneously assembles to form positively charged nanoparticles composed of 7 copies of the NP fused to OVX313.

Animal studies have demonstrated the ability of OVX836 to elicit humoral and cellular immunity – including tissue-resident and long-lasting CD8+ T-cells in the lungs - as well as protection in mice and ferrets against influenza A and B challenges (16, 18).

The results of a Phase 1, randomized, placebo-controlled clinical study have recently been published (19). When administered by the intramuscular (IM) route, OVX836 was safe and well-tolerated at 30µg, 90µg and 180µg, administered as a two-dose schedule with one month interval. A single injection of OVX836 was able to significantly increase the number of NP-specific T-cells at Day 8 and titers of anti-NP IgG at Day 29. The second vaccination (28 days after the first) did not amplify the immune response. There was no clear dose-effect relationship between 30µg, 90µg and 180µg. However, a dose of 30µg was considered potentially insufficient to induce a strong and persistent immune response, especially in populations with weakened immunity, such as older adults. Therefore, OVX836 dose levels of 90µg and 180µg were selected to be further evaluated in a Phase 2a study (ClinicalTrials.gov Identifier: NCT04192500; EudraCT number 2019-002939-28; IND number 19276), the object of the current paper.

## Materials and Methods

This randomized, reference-controlled, double-blind Phase 2a study was performed at one single center, the Center for Vaccinology (CEVAC, Ghent University and University Hospital, Ghent, Belgium), in accordance with Good Clinical Practice. It was approved by the Ethics Committee of the Ghent University Hospital and by the Belgian Federal Agency for Medicines and Health Products (FAMHP). All participants gave their written informed consent.

Three hundred healthy adult men and women, aged 18-65 years were included in this study.

The exclusion criteria were body mass index ≥35 kg/m², active smoking (more than 10 cigarettes/day), and pregnancy or unwillingness to practice birth control. Any known or suspected immunodeficient conditions, autoimmune disorders or chronic diseases, or presence of an acute febrile illness on the day of vaccination led to exclusion, as well as previous influenza vaccination within 6 months before screening, vaccination within 3 or 1 month prior to the day of study vaccination for live attenuated or inactivated vaccines, respectively. Subjects taking treatments that could affect the immune response (systemic corticosteroids, cytotoxic drugs, anti-inflammatory drugs and other immunomodulatory drugs) were also excluded.

Participants (N=300) were stratified in two age strata (18-49 year-old subjects representing 65-70% and 50-65 year-old subjects representing 30-35% of the overall cohort) and randomized (1:1:1) into 3 groups of 100 subjects who received a single IM injection (deltoid muscle of the non-dominant arm) of either OVX836 at 90µg (300 µg/mL; 0.3 mL), OVX836 at 180µg (300 µg/mL; 0.6 mL) or a quadrivalent seasonal influenza subunit vaccine (Influvac Tetra™, Mylan; 2019-2020 season, 0.5mL dose) (**Supplementary Figure 1**). Influvac Tetra is not expected to contain NP (20) and was therefore chosen as placebo-like comparator for the immunogenicity analysis, without the ethical constraints of a real placebo. Safety monitoring consisted of a 7-day post-vaccination follow-up period for solicited local (injection site pain, redness and swelling) and systemic signs and symptoms (fatigue, headache, arthralgia, malaise, myalgia and fever [oral temperature]) using an electronic diary (eDiary), a 28-day follow-up period for unsolicited adverse events (AEs), and a 180-day follow-up period for serious AEs (SAEs) and ILI to detect potential Vaccine-Associated Respiratory Disease. The grading of solicited signs and symptoms was performed in accordance with FDA guidance (https://www.fda.gov/regulatory-information/search-fda-guidance-documents/toxicity-grading-scale-healthy-adult-and-adolescent-volunteers-enrolled-preventive-vaccine-clinical), with slight modifications. Subjects had to visit the clinical site on Day 1 (injection day, visit 1) and on Days 8 (visit 2), 29 (visit 3) and 180 (visit 4, end of study visit) post-injection. Missed visits due to containment measures for the COVID-19 pandemic implemented at the start of the study (on 19 March 2020), were replaced by duly documented phone contacts. PBMC samples were collected at each visit to measure NP-specific T-cell responses (interferon-gamma [IFNγ] enzyme-linked immunospot assay [ELISPOT], and CD4+ and CD8+ T-cells expressing IFNγ, interleukin-2 [IL-2] and/or tumor necrosis factor alpha [TNFα] by flow cytometry, according to the gating strategy as described in **Supplementary Figure 2**). Immunoassays are described in the **Supplementary Methods**.

Subjects presenting with influenza-like illness (ILI) were requested to return to the investigational site as soon as possible for laboratory confirmation of influenza infection (RT-PCR assay performed on nasopharyngeal [NPh] swabs) and to monitor the severity of the disease until resolution of symptoms using the Flu-PRO^®^ questionnaire (21). Due to unavailability of NPh swab samples in some subjects as a result of containment measures for the COVID-19 pandemic, hemagglutination inhibition assays (HAI specific of A/H1N1/Brisbane/02/2018 and A/H3N2/Kansas/14/2017 strains, two strains representative of those that circulated most during 2019-20 influenza season) were performed on Day 1 and Day 180 samples of OVX836 groups as additional means to identify influenza infection as causal agent of the ILI episode. A four-fold increase in HAI titers between Day 1 and Day 180 was considered indicative of an influenza infection. In the Influvac Tetra cohort, where the vaccine induced a rise in HAI titers, a four-fold increase between Day 1 and Day 180 in anti-NP IgG was considered as a serological marker of influenza infection (22, 23).

The epidemiological data from Sciensano (Belgian Institute for Public Health), namely the percentage of laboratory-confirmed flu positive samples (**Supplementary Figure 3**), were used to define the flu season as the period between 02 December 2019 and 09 March 2020 (weeks during which percentage of lab-confirmed influenza cases was above 20%) (24).

The study was designed to detect a difference of 2.3 between mean ratios (Day 8/Day 1; primary endpoint) for Influvac Tetra (1.0) and for OVX836 (3.3) in the NP-specific IFNγ SFC counts measured by ELISPOT, assuming that the common standard deviation was 4.0, using a two group t-test with a 0.017 (Bonferroni’s correction for three comparisons) two-sided alpha significance level. A sample size of 83 subjects in each group would have 90% power to detect such a difference. To account for potential dropouts, a total of 100 subjects per group were included in the study.

SAS (Version 9.4) was used for the statistical analyses. Descriptive statistics were used to summarize all relevant parameters and the balance between the three treatment groups at baseline was confirmed. Continuous variables were analyzed using analyses of variance for repeated measures (PROC MIXED) with the factor ‘time’, ‘treatment group or age strata’, and interaction of both factors as fixed factors. When justified, the appropriate *post-hoc* intergroup and intragroup comparisons were performed. Discrete variables were analyzed using logistic regression analysis (PROC LOG) with a multiplicity correction for *post-hoc* pairwise comparisons. Kaplan-Meier survival analysis and log-rank tests were used to compare the time elapsed between vaccination and occurrence of ILIs in the three treatment groups.

## Results

### Demographic and Baseline Characteristics

Three hundred subjects (208 aged 18-49 years and 92 aged 50-65 years) were vaccinated between 11 December 2019 and 09 March 2020 (100, 101 and 99 with OVX836 90µg, OVX836 180µg and Influvac Tetra, respectively); all were included in the modified intention-to-treat (mITT) cohort (**Supplementary Figure 4**). All subjects completed the study and had samples taken on Day 8 (primary endpoint). Due to the COVID-19 containment measures (replacement of visits by phone contacts), samples for the immunogenicity analyses on Day 29 could only be taken in 227 participants (per protocol [PP] cohort: 78, 73 and 76 in the OVX836 90µg, OVX836 180µg and Influvac Tetra group, respectively).

Demographic data and baseline characteristics of the 300 study participants are shown in **Supplementary Table 1**.

### OVX836 Induced Significant Increase of NP-Specific Cellular Immune Responses and Anti-NP IgG Titers

In the 227 subjects of the PP cohort, the mean ± SD (median) Day 8/Day 1 NP-specific IFNγ SFC ratio was 2.92 ± 3.04 (1.83) in the OVX836 90µg group, 2.66 ± 2.21 (1.93) in the OVX836 180µg group and 1.51 ± 1.55 (1.03) in the Influvac Tetra group. The overall mean difference between groups was significant (p<0.001; ANOVA). Planned *post-hoc* tests revealed significant differences between OVX836 90µg and Influvac Tetra (p<0.001, Bonferroni’s test), as well as between OVX836 180µg and Influvac Tetra (p<0.01), without significant difference between the two OVX836 dose levels on this parameter. The primary endpoint of the study was therefore met.

**Figure 1:**
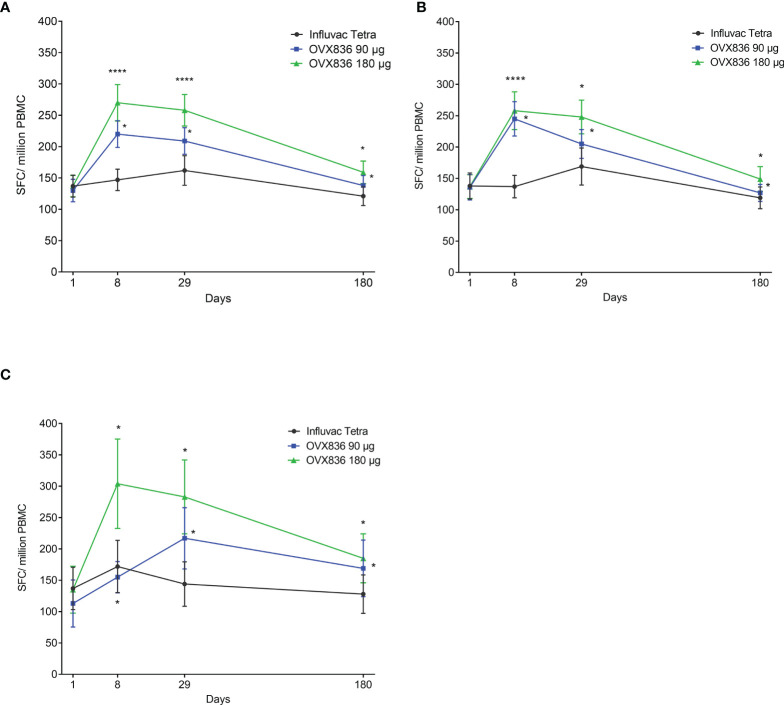
Evolution over time of the number of nucleoprotein (NP)-specific interferon-gamma (IFNγ) spot forming cells (SFCs) per million of peripheral blood mononuclear cells (PBMC) between baseline (Day 1 – pre-vaccination) and Day 180 (post-vaccination) in the three treatment groups (Influvac Tetra™, OVX836 90µg and OVX836 180µg; per protocol cohort). Results are shown as mean ± standard error; paired comparisons between each timepoint post-vaccination and baseline (Day 1): *p<0.05; ****p<0.0001. Panel **(A)** corresponds to all subjects, Panel **(B)** to subjects aged 18-49 years and Panel **(C)** to subjects aged 50-65 years.

The NP-specific IFNγ SFC responses in all subjects, subjects aged 18-49 years and subjects aged 50-65 years are shown in **Figure 1**. Differences between OVX836 180µg and Influvac Tetra were significant (p<0.05) on Days 8 and 29 in all subjects and in the two separate age strata. Subjects aged 50-65 years showed a higher response to OVX836 180µg than to OVX836 90µg.

The anti-NP IgG responses are shown in **Supplementary Figure 5**. There was a significant increase in titers on Day 8, peaking at Day 29 and lasting up to Day 180 with OVX836 at both doses compared to Influvac Tetra. In subjects aged 18-49 years no dose effect of OVX836 on anti-NP IgG responses was observed throughout the observation period. In subjects aged 50-65 years OVX836 at 180µg elicited a higher anti-NP IgG response only on Day 29 as compared to 90µg dose. The persistence of the responses did not differ between older and younger subjects.

No clear correlations were found between the NP-specific humoral and cellular responses (SFCs and CD4+ T-cell responses), all correlation coefficients being inferior to 0.5 (data not shown).

### OVX836 Vaccination Increased Polyfunctionality of CD4+ T-Cells

**Figure 2:**
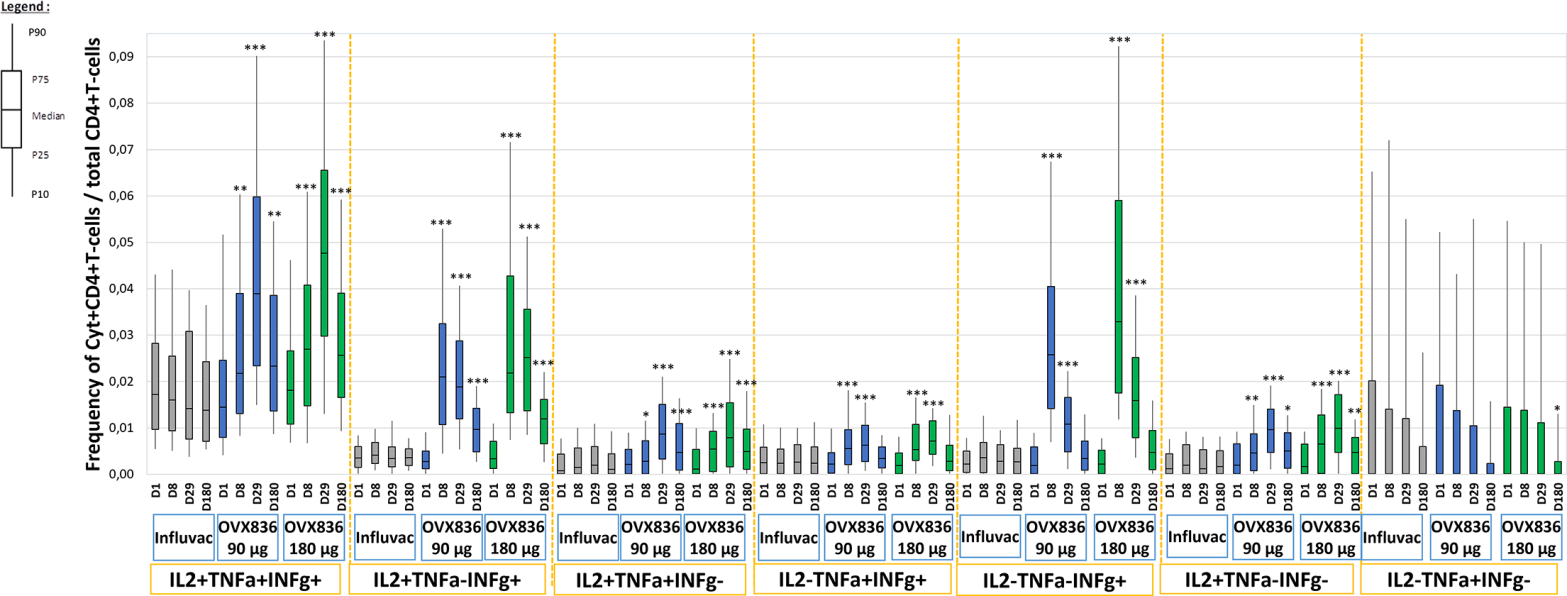
Frequencies of nucleoprotein (NP)-specific CD4+ T-cells identified *in vitro* as expressing the combinations of cytokines: IFNγ, IL-2 and TNFα at pre-vaccination, Day 1 (D1), Day 8 (D8), Day 29 (D29) and Day 180 (D180) in the three treatment groups (Influvac Tetra™, OVX836 90µg and OVX836 180µg; modified intention-to-treat cohort). Results are shown as box plot with the horizontal central bar corresponding to the median, limits of the box corresponding to percentiles 25 and 75, and vertical bars corresponding to percentiles 10 and 90. Intragroup differences: *p<0.05, **p<0.01 and ***p<0.0001; paired t tests versus baseline. Main intergroup differences (global ANOVA with p value <0.05 at each timepoint and *post-hoc* t tests between OVX836 90 µg and OVX836 180 µg with p values <0.05) were as follows: - IL2+/TNFα+/IFNγ+: Day 8 global p=0.012, Day 29 global p<0.0001, Day 180 global p<0.0001. - IL2+/TNFα-/IFNγ+: Day 8 global p<0.0001, Day 29 global p<0.0001 (OVX836 180 µg versus OVX836 90 µg p=0.008), Day 180 global p<0.0001 (OVX836 180 µg versus OVX836 90 µg p=0.026). - IL2+/TNFα+/IFNγ-: Day 8 global p=0.036, Day 29 global p<0.0001, Day 180 global p<0.0001. - IL2-/TNFα+/IFNγ+: Day 8 global p<0.0001, Day 29 global p=0.001. - IL2-/TNFα-/IFNγ+: Day 8 global p<0.0001 (OVX836 90 µg versus OVX836 180 µg p=0.002), Day 29 global p<0.0001 (OVX836 90 µg versus OVX836 180 µg p<0.0001). - IL2+/TNFα-/IFNγ-: Day 8 global p<0.0001 (OVX836 90 µg versus OVX836 180 µg p=0.026), Day 29 global p<0.0001, Day 180 global p=0.003. - IL2-/TNFα+/IFNγ-: No statistically significant differences (global p values>0.05).

The percentage of CD4+ T-cells expressing at least IFNγ in all subjects, and split in subjects aged 18-49 years and aged 50-65 years, is shown in **Supplementary Figure 6**. The percentage of NP-specific CD4+ T-cells with the different profiles of expression of IFNγ, IL-2 and TNFα in the three treatment groups and at the different timepoints is shown in **Figure 2**. The baseline level was low (median ranging around 0.005%) in the three groups and no increase was observed in the Influvac Tetra group. Both dose levels of OVX836 induced a significant CD4+ response on Days 8 and 29. In subjects aged 18-49 years the peak response was observed on Day 8 whereas in subjects aged 50-65 years (**Supplementary Figure 6**) the response peaked on Day 29. The CD4 response was predominantly polyfunctional with most CD4+ T-cells co-expressing three (IL-2/TNFα/IFNγ) and two (IL-2/IFNγ) cytokines, in addition to CD4+ cells expressing IFNγ only. The evolution over time of polyfunctional CD4+ T-cells was relatively similar in subjects aged 18-49 years and in subjects aged 50-65 years (data not shown).

### OVX836 Increased CD8+ T-Cells Expressing at least IFNγ in Subjects With Low Baseline Values

In the mITT cohort, no significant increase of CD8+ T-cells was observed in any of the groups, including those given OVX836. It must be noted that baseline (Day 1) values were high (median value ranging between 0.112% and 0.121%), especially for subjects vaccinated after 30 January 2020, which may be linked to the active circulation of the influenza virus (**Supplementary Figure 7A**). In subjects belonging to the lowest quartile of the CD8+ response at baseline (most likely the ones with the lowest probability of recent exposure to influenza preceding vaccination), the median percentage of NP-specific CD8+ T-cells expressing IFNγ increased significantly (from 0.03% on Day 1 to 0.06% on Day 8; p=0.020) in the OVX836 180µg group only (**Supplementary Figure 7B**).

### A Dose-Response Effect Was Observed With OVX836 180µg Performing Better Than 90µg

**Figure 3:**
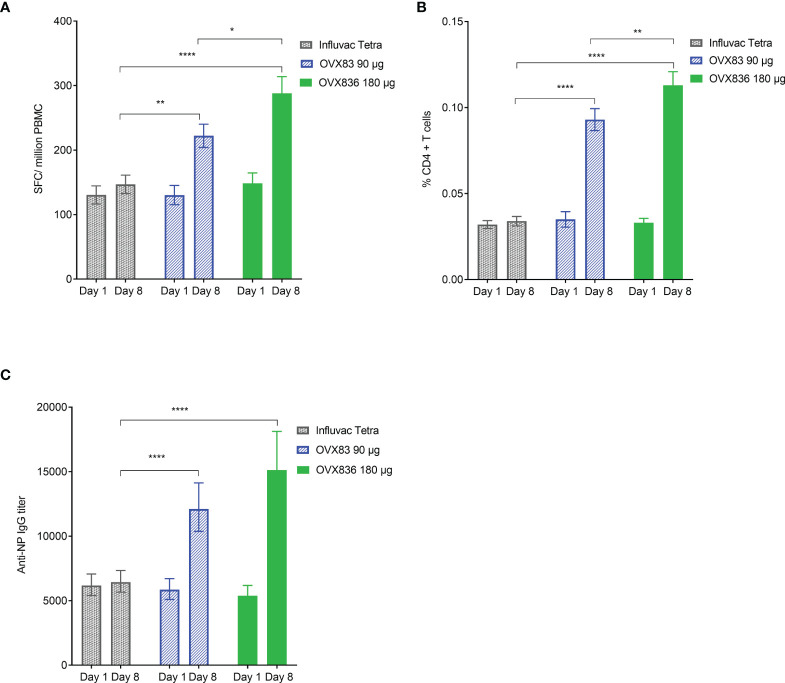
Panel **(A)** Number of nucleoprotein (NP)-specific interferon-gamma (IFNγ) spot forming cells (SFCs) per million peripheral blood mononuclear cells (PBMC) at baseline (Day 1 – pre-vaccination) and Day 8 (post-vaccination) in the three treatment groups (Influvac Tetra™, OVX836 90µg and OVX836 180µg; modified intention-to-treat cohort after elimination of two outlier subjects with very high values at baseline). Results are shown as mean ± standard error; intergroup comparisons: *p<0.05; **p<0.01; ****p<0.0001. Panel **(B)** Percentage of NP-specific CD4+ T-cells positive for at least IFNγ at baseline (Day 1 – pre-vaccination) and Day 8 (post-vaccination) in the three treatment groups (modified intention-to-treat cohort). Results are shown as mean ± standard error; intergroup comparisons: **p<0.01; ****p<0.0001. Panel **(C)** Anti-NP immunoglobulin G (IgG) titers at baseline (Day 1 – pre-vaccination) and Day 8 (post-vaccination) in the three treatment groups (modified intention-to-treat cohort). Results are shown as geometric mean titers ± 95% confidence interval; intergroup comparisons: ****p<0.0001.

The OVX836 dose-effect relationship in terms of SFCs, CD4+ T-cell expressing at least IFNγ and anti-NP IgG responses is illustrated, in the mITT, in **Figure 3**. No differences were found between the three groups at baseline (Day 1). On Day 8, a statistically significant (p<0.05 to p<0.0001) increase in the three parameters was measured with OVX836 at both doses versus Influvac Tetra. The response to OVX836 180µg was significantly higher compared to OVX836 90µg for SFCs (p=0.006) and CD4+ T-cell expressing IFNγ (p=0.007) but not significant for anti-NP IgG (p=0.085).

### OVX836 Was Safe and Presented a Reactogenicity Profile Similar to Influvac

**Figure 4:**
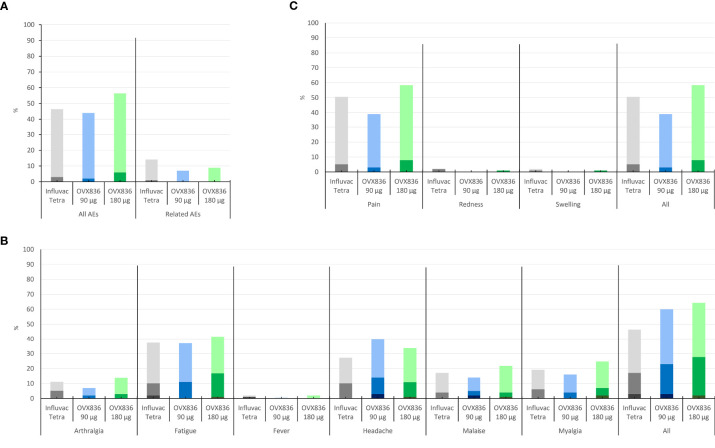
Panel **(A)** Percentage of subjects reporting mild (light color) or moderate (dark color) solicited local signs during the 7-day post-vaccination period in the three treatment groups (Influvac Tetra™, OVX836 90µg and OVX836 180µg; modified intention-to-treat cohort corresponding to the safety cohort). Panel **(B)** Percentage of subjects reporting mild (light color), moderate (intermediate color) or severe (dark color) solicited systemic symptoms during the 7-day post-vaccination period in the three treatment groups and safety cohort. Panel **(C)** Percentage of subjects reporting unsolicited adverse events (AEs) (light color) and severe AEs (grade 3; dark color), overall and considered related to the vaccine by the investigator, during the 28-day post-vaccination period, in the three treatment groups and safety cohort.

Mild to moderate solicited local signs and symptoms (almost exclusively local pain at the injection site) were reported by 39%, 58% and 51% of subjects in the OVX836 90µg, OVX836 180µg and Influvac Tetra groups, respectively (**Figure 4A**). Systemic signs and symptoms (all grades) were reported by 60%, 64% and 46% of subjects and signs and symptoms graded “severe” by 3%, 2% and 3% of subjects in the OVX836 90µg, OVX836 180µg and Influvac Tetra groups, respectively (**Figure 4B**). In the OVX836 180µg group, 4 severe systemic signs and symptoms were reported by the same subject concomitantly with a RT-PCR-confirmed influenza episode. The mean duration of all solicited signs and symptoms taken together was 1.8 ± 3.2 days in the OVX836 90μg group, 1.9 ± 3.4 days in the OVX836 180μg group and 2.5 ± 4.1 days in the Influvac Tetra group.

Unsolicited AEs considered related to the treatment were reported by 7%, 9% and 13% of subjects in the OVX836 90µg, OVX836 180µg and Influvac Tetra groups, respectively (**Figure 4C**). The most frequent AEs, affecting at least 5% of subjects were ILIs, Upper Respiratory Tract Infection, Headache, Oropharyngeal Pain and Lymphadenopathy (**Supplementary Table 2**). Overall, 5 SAEs were reported in 2 subjects of the OVX836 90µg group (Appendicitis and Sinus tarsi syndrome), 1 subject of the OVX836 180µg group (Thyroid cancer), and 2 subjects of the Influvac Tetra group (Radius fracture and Intervertebral disc protrusion). None of the reported SAEs was considered related to the vaccine and none of them led to study discontinuation. The reported cases of ILI did not suggest any disease enhancement by the vaccine.

### A First Efficacy Signal Observed With OVX836 at 180µg on the Reduction of ILI Episodes

**Figure 5:**
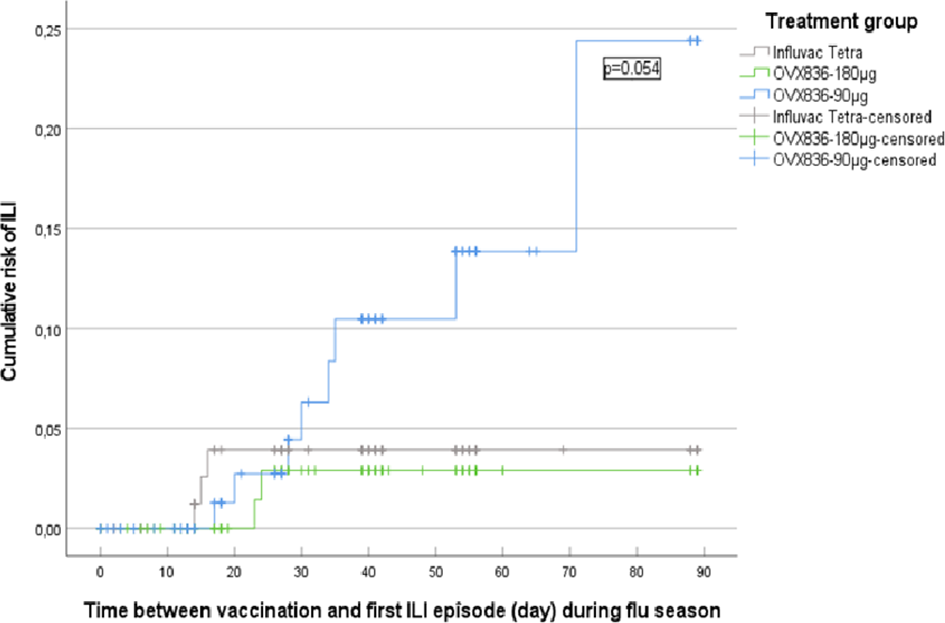
Kaplan-Meier survival analysis of the cumulative risk of influenza-like illness (ILI) as a function of time between vaccination and first ILI episode (day) during the influenza season (defined as the period between 02 December 2019 and 09 March 2020), from 14 days post-vaccination onward, in the three treatment groups (Influvac Tetra™, OVX836 90µg and OVX836 180µg; modified intention-to-treat cohort corresponding to the safety cohort). Log-rank test showed a p=0.054 for the comparison between Influvac Tetra and OVX836 90µg.

Kaplan-Meier survival analyses were used to evaluate the cumulative hazard of ILIs as a function of post-vaccination time during the influenza season (defined by Sciensano – former Belgian Scientific Institute of Public Health, as the period between 02 December 2019 and 09 March 2020) (24). A first analysis took into account all ILIs occurring during the 2019-2020 flu season (**Supplementary Figure 8**). The comparison between the three treatment groups was non-significant (p=0.325; log-rank test). A second analysis took into account the ILIs occurring during the same period but only from 14 days post-vaccination onward based on the assumption that the immune response will require some time after vaccination to reach protective levels. Thirteen ILIs occurred from 14 days post-vaccination onward: 8 in the OVX836 90µg group, 2 in the OVX836 180µg group and 3 in the Influvac Tetra group (**Figure 5**). There was a trend for a global difference between the three groups (p=0.088) and for a difference between OVX836 180µg and OVX836 90µg (p=0.054). The difference between OVX836 90µg and Influvac Tetra was not significant (p=0.130). Among these 13 ILIs, 4 (31%) were lab-confirmed influenza cases, either by RT-PCR and/or HAI seroconversion (4-fold increase) in the OVX836 groups, or anti-NP IgG seroconversion (4-fold increase) in the Influvac Tetra group: 2 in subjects of the OVX836 90µg group (1 confirmed by RT-PCR and 1 confirmed by 4-fold HAI titer increase), 0 in subjects in the OVX836 180µg group and 2 in subjects of the Influvac Tetra group (confirmed by 4-fold anti-NP IgG increase).

Twenty-three (24) ILI cases were observed during the thirteen days consecutive to vaccination, the period wherein the protective effect of the vaccine is assumed to be still very low or absent. These cases were used to examine whether an association exists between the pre-vaccination levels of NP-specific IFNγ SFCs and CD4+ T-cells (expressing at least IFNγ) and the risk of developing an ILI in the overall population (three groups pooled together). NP-specific IFNγ SFCs were 150 ± 184 and 69 ± 49 SFC/million PBMCs (p<0.001) in subjects who did not report any ILI and subjects who reported at least one ILI, respectively. A significant difference was also found for NP-specific CD4+ T-cells expressing at least IFNγ (0.034 ± 0.034% versus 0.022 ± 0.011%; p<0.0001) (**Supplementary Table 3**).

## Discussion

A single IM injection of 90µg or 180µg of the OVX836 vaccine candidate was safe and well-tolerated. This confirms the observations made during the Phase 1 study (19). The overall safety profile, in terms of nature, intensity and duration of local and systemic signs and symptoms, was very similar to that of the tetravalent reference vaccine Influvac Tetra. No severe adverse events attributable to OVX836 were observed, suggesting that the maximum tolerated dose was not reached in this study.

A single dose of OVX836 at 90µg or 180µg was able to significantly increase the NP-specific IFNγ SFCs, the polyfunctional and IFNγ secreting CD4+ T-cells and the anti-NP IgG level in both the 18-49 years-old and the 50-65 years-old subjects. The persistence of these humoral and cellular immune responses was comparable in both age groups. Administration of the control vaccine, Influvac Tetra, a subunit seasonal influenza vaccine devoid of NP, did not significantly increase the NP-specific immune responses in vaccinated subjects. The primary objective of the trial, namely the demonstration of superiority of the NP-specific IFNγ SFC response to OVX836 (at both dose levels) at Day 8 in the whole cohort, compared to Influvac Tetra, was thus achieved. The NP-specific immunity observed at baseline, the rapid rise of anti-NP IgG in the 18-49 years-old subjects already one week after a single dose of OVX836 and the polyfunctionality of CD4+ T-cells (IL-2+TNFα+IFNγ+) observed at baseline are consistent with a recall of pre-existing NP-specific immunity in the subjects, induced by preceding exposure to the influenza virus.

Overall, OVX836 at 180µg performed better than OVX836 at 90µg, even though OVX836 vaccination at both dose levels was able to increase NP-specific responses. The dose-effect level was significant for the NP-specific IFNγ SFC response and for the frequency of CD4+ T-cells expressing IFNγ at Day 8. It was also observed in the older subgroup, in which only the 180µg dose was able to significantly increase the NP-specific SFC response and the CD4+ T-cells on Day 8.

Flow cytometric analysis of T-cells induced after OVX836 vaccination showed that polyfunctional NP-specific CD4+ T-cells, co-expressing IFNγ, TNFα and IL-2, already present at lower level at baseline, increased significantly at Day 8 and even more at Day 29 after OVX836 vaccination, both in young and older subjects. Interestingly, this increase of polyfunctional CD4+ T-cells persisted until the end of the study period (Month 6), showing that the candidate vaccine induced/boosted NP-specific CD4+ T cells expressing functionally relevant anti-viral cytokines, i.e., IFNγ and TNFα. This is of importance, since polyfunctional cellular responses have been reported to be associated with protection against viral and bacterial infections (25–27).

An increase of NP-specific CD8+ T-cells was not observed in the OVX836 vaccinated subjects. The intense circulation of influenza virus during the recruitment period of the study participants appears to have led to high baseline levels of NP-specific immunity, in particular of CD8+ T-cells. This may have jeopardized the detection of ensuing changes of signals. This assumption is supported by the fact that when the lowest quartile of CD8+ responders at baseline was analyzed separately, a significant CD8+ response could be observed on Day 8 for the OVX836 180µg dose level, which was not the case in the OVX836 90µg and Influvac Tetra groups. Another hypothesis could be that the vaccine antigen may not be effectively cross-presented to induce CD8+ T-cell responses against NP.

OVX836 induced significant increases in anti-NP IgG, irrespective of the dose administered. Although not being neutralizing as NP is an internal antigen of the influenza virus, these antibodies may play a role in viral clearance by contributing to the elimination of infected cells (28), *via* antibody-dependent cellular cytotoxicity (29), immune complex natural killer cell priming (30) or other mechanisms (31).

Both the CD4+ T-cell and the anti-NP IgG responses were persistent and remained significantly higher than baseline at Day 180. The IFNγ SFC response remained significantly higher than the baseline value 6 months after the immunization when excluding the subjects situated in the highest quartile who had probably been exposed to influenza before vaccination (Day 1). As already observed in Phase 1 study (19), the OVX836-induced T-cells peaked one week after vaccination and decreased afterwards while remaining above baseline at Day 180. The kinetics of the vaccine-induced NP-specific T-cell response is consistent with that observed after influenza infection and corresponds to the expansion and contraction phases described for T-cell responses (10, 32–34). The disappearance of T-cells from the blood compartment coincides with the postulated sequestration of some of these cells into functional memory cells localized in secondary organs or non-lymphoid organs. These cells are known to play a critical role in the fight against subsequent infections (35).

The level and evolution over time of immune responses was similar for both age-groups (18-49 and 50-65 years old) for almost all immune parameters, with sustained immune responses for both age groups. However, the CD4 IFNγ+ and anti-NP IgG responses peaked later in the 50-65 years old group. Similarly, the 90µg dose-level, but not the 180µg dose-level, triggered a total T-cell response which peaked at Day 29 for subjects aged 50-65 years versus Day 8 for the younger age group. The consistency of this response pattern will be examined in future studies that will also include adults older than 65 years.

Even though the study was not designed to show clinical efficacy, ILI follow-up during the influenza season allowed to make interesting observations. During the first 13 days after vaccination, no difference was observed in occurrences of ILIs between the three groups. This period likely coincides with the time the vaccines need to mount a protective immune response. Although the NP-specfic CD4+ response measured in the peripheral blood peaked at Day 8 in younger adults, it may take more time to reach a protective effector response in the respiratory tissues. In addition, ILI symptoms usually appear 1-4 days after exposure to the virus (incubation period). However, from 14 days post-vaccination onward until the end of flu season, fewer ILIs were observed in the OVX836 180µg and Influvac Tetra groups as compared to OVX836 90µg. It is tempting to consider these differences as a signal of vaccine effect but a large efficacy trial is needed to confirm this trend.

We observed an association of pre-vaccination levels of IFNγ SFC and CD4+ T-cells (producing at least IFNγ) with a lowered risk for developing an ILI in the first few days after vaccination (i.e., before any vaccines were effective, three groups pooled together) in the whole vaccinated cohort. This corroborates the observations made earlier by Wilkinson et al. (32), correlating preexisting influenza-specific CD4+ with disease protection against influenza challenge in humans. This is also in line with the observations made by some of the authors, in a collaboration with the company hVivo, which pointed to the threshold of 100 SFC/10^6^ PBMCs in the association with symptoms during the challenge study (36). Interestingly, only in the 180µg group did the levels of NP-specific IFNγ SFC triggered by OVX836 remain above the thresholds of 100 SFC/10^6^ PBMCs (36) for more than 50% of the subjects, a threshold considered as predictive of an efficacy against PCR-confirmed symptomatic influenza in adults using the same assay as the one used in this study.

Several vaccines aiming to induce T-cell responses against NP have been evaluated in clinical trials. FLU-V, a vaccine composed of an adjuvanted pool of peptides encoding for epitopes from NP and other influenza proteins successfully demonstrated efficacy in a challenge study (37) and a signal of efficacy in a field trial in the range of the signal described in this paper (38), but also cases of severe local inflammation likely attributable to the adjuvant. Other approaches targeting T-cell responses against NP using different technologies have failed to demonstrate efficacy in clinical trials (NCT03450915 and NCT03883113).

The first limitation of this study is the fact that vaccination occurred during the influenza season, which had an impact on several immune parameters (e.g. CD8+ T-cells). A second limitation is the interference of the COVID-19 pandemic which prevented taking 20% of the Day 29 samples and performing PCRs after 17^th^ March 2020, even though the primary endpoint was secured as all subjects were recruited before the COVID-19 epidemic peak. A third limitation is the fact that the study was not designed and powered to demonstrate efficacy, so that the efficacy signal observed cannot be conclusive.

In conclusion, these results warrant further evaluation of the safety and immunogenicity of higher doses of OVX836 (300µg and 480µg in addition to 180µg) in order to select the best dose for a Phase 2b trial designed and powered to confirm the signal of efficacy on laboratory-confirmed flu cases.

## Data Availability Statement

The datasets presented in this article are not readily available because the dataset is confidential and proprietary to OSIVAX. Requests to access the datasets should be directed to alevert@osivax.com.

## Ethics Statement

The studies involving human participants were reviewed and approved by Ethics Committee of the Ghent University Hospital in Belgium. The patients/participants provided their written informed consent to participate in this study.

## Author Contributions

IL-R was the Principal Investigator of this study, GW was responsible for the cell-mediated immunoassays at the CEVAC laboratory, FB was the study coordinator at CEVAC, CM was co-investigator, GL-R was scientific advisor and member of the Clinical Advisory Committee of the sponsor, JT (sponsor’s Clinical Operations Director), DG-G (sponsor’s Chief Business Development and Strategy Officer), PW (sponsor’s Chief Medical Officer), PM (sponsor’s Head of Clinical Immunology), JC (sponsor’s Head of Immunology Research & Development), JB (sponsor’s Head of Biometry and Medical Writing), AV (sponsor’s Chief Executive Officer) and FN (sponsor’s Chief Development Officer) contributed to the design, management, medical monitoring, analyses and reporting of this clinical study. All authors contributed to the article and approved the submitted version.

## Funding

This project has received funding from Bpifrance (grant nr DOS0105407/00) and from the European Union’s Horizon 2020 Research and Innovation Program under grant agreement Nr 961112.

## Conflict of Interest

JT, DG-G and JC are employees of Osivax. PW, PM, JB and GL-R are consultants who received fees from Osivax. AV and FN are shareholders and executive members of Osivax.

The remaining authors declare that the research was conducted in the absence of any commercial or financial relationships that could be construed as a potential conflict of interest.

## Publisher’s Note

All claims expressed in this article are solely those of the authors and do not necessarily represent those of their affiliated organizations, or those of the publisher, the editors and the reviewers. Any product that may be evaluated in this article, or claim that may be made by its manufacturer, is not guaranteed or endorsed by the publisher.
