# Supplementary material for: Randomized, Double-Blind, Reference-Controlled, Phase 2a Study Evaluating the Immunogenicity and Safety of OVX836, A Nucleoprotein-Based Influenza Vaccine
Source: Front Immunol. 2022 Apr 7;13:852904. doi: 10.3389/fimmu.2022.852904 (PMC9022189; doi:10.3389/fimmu.2022.852904)
Supplement: Supplementary file 1 [file DataSheet_1.docx]

Supplementary Material

# Supplementary Methods

## ELISA for anti-NP serum IgG

The method is an indirect ELISA including the following steps: 1) coating of the wells of a 96-well plate with NP (Osivax R&D batch), 2) blocking of non-specific binding sites with bovine serum albumin, 3) incubation with the clinical samples after two-fold serial dilutions (first dilution 1:800), 4) addition of the secondary anti-human IgG antibody conjugated to horse radish peroxidase (provider), and 5) revelation of the signal using 3,3',5,5'-tetramethyl-benzidine (TMB, Sigma) substrate. The anti-NP IgG titer for a serum sample was defined as the highest tested dilution that gives an optical density >2 fold the signal of the negative control tested on each plate. The method was qualified. A reference positive quality control (QC+) was obtained by pooling 50 sera from healthy adults and a negative control (QC-) was obtained by pooling 4 serum from very young children (assumed not to have been exposed to influenza based on the very weak anti-NP signal). Two additional controls (QC low and QC high) were prepared from the reference and negative controls and also used for the qualification. The method was qualified for within-run (n≥5) and between-run (n≥5, on different days with 2 operators) precision and showed good performance (coefficient of variation [CV] <20% on QC low and medium; QC+ titer: 6400 ± 1 dilution step). The stability of the QC samples upon 3 freeze-thaw cycles was also checked. The QC samples were used in parallel to clinical samples, to validate each plate according to predefined criteria.

## Peripheral Blood Mononuclear Cell (PBMC) processing

50 mL of blood were collected by venous puncture at the selected time points in heparin coated blood collection tubes (Becton Dickinson Vacutainer tubes of 10 mL coated with lithium heparin). After 1:2 dilution in Hanks buffered salt solution (HBSS) buffer, PBMCs were isolated by density gradient centrifugation (Ficoll-Hypaque – provider), washed twice in HBSS, suspended in freezing solution (10% dimethyl sulfoxide (DMSO)/90% fetal calf serum (FCS) v/v), frozen at a concentration of minimum 5 and maximum 20 million cells/mL and stored in liquid nitrogen until use.

## ELISPOT assay on PBMCs for the anti-NP T-cell response

An ELISPOT method was used to measure the number of PBMCs that secreted IFN-γ upon in vitro re-stimulation with NP (NP-specific T-cells). After PBMC thawing, resuspension in culture medium, and overnight resting, 2x10^5^ PBMCs/well were incubated for 24 hours with 4μg per peptide/mL of a NP peptide pool from influenza A/WSN/33 (H1N1) strain, in triplicate. The pool was composed of 122 15-mer peptides overlapping by 11 amino acids, covering the NP sequence. As a negative control, cells were incubated in the same conditions with culture medium supplemented with 0.6% DMSO. The average number of spot-forming cells (SFC) in the negative control was subtracted from the average number of SFC for the test wells. In doing so, the final result was reported as SFC per million PBMCs for each clinical sample. Samples for which viability post-resting was below 70% were not included in the results analysis. The ELISPOT assay was qualified. The method showed a good performance for the evaluation of Influenza virus NP-specific T-cell responses, with acceptable within- and between- run precision on the samples used for qualification (CV <30%, for a level of signal of ≈ 100-200 SFCs/million PBMCs).

## Flow cytometry – Intracellular cytokine staining (ICS) for PBMC

After thawing of PBMC samples, the cells were washed with culture medium (RPMI supplemented with MEM non-essential amino acids, sodium pyruvate, Pen/Strep, L-glutamine β-mercaptoethanol and fetal calf serum), then incubated in vitro with the relevant antigen versus corresponding medium in the presence of costimulatory antibodies to CD28 and CD49d for two hours. Then Brefeldin A, a protein secretion blocker, was added for the subsequent overnight culture. This step ensured the inhibition of cytokine secretion and its accumulation in the expressing cells. The next day, the cells were stained using fluorochrome-conjugated antibodies to phenotypic (CD3, CD4 and CD8) markers and cytokine (IFNγ, IL-2 and TNFα) markers. All details concerning the fluorochrome-conjugated antibodies can be found in the table below. The cells were then analyzed by flow cytometry (BD LSR Fortessa X-20, FlowJo v9.9.6).

| **Reagent specificity** | **Clone** | **Conjugated dye** | **Catalog** | **Provider** |
| --- | --- | --- | --- | --- |
| CD3 | UCHT1 | AF700 | 557943 | Becton DickinsonD |
| CD4 | SK3 | PerCP | 345770 | Becton DickinsonD |
| CD8 | SK1 | APC-H7 | 641400 | Becton DickinsonD |
| IFNγ | 4S.B3 | FITC | 554551 | Becton DickinsonD |
| TNFα | Mab11 | PE-Cy7 | 557647 | Becton DickinsonD |
| IL-2 | MQ1-17H12 | APC | 554567 | Becton DickinsonD |

The relevant antigen and the stimulation conditions were the same as those used in the ELISPOT assay. The concentration of stimulation was 4μg per peptide/mL and negative control including 0.3% DMSO was tested for each sample.

The NP-specific CD4+ and CD8+ T cells were determined by flow cytometry as the CD3+CD4+ and CD3+CD8+ events expressing at least one cytokine or a combination of cytokines among IFNγ, IL-2 and TNFα after in vitro stimulation with the relevant antigen subtracted by the corresponding signal of the same sample obtained after in vitro stimulation with medium + DMSO (blank), according to the illustrative example depicted in the Supplementary Figure 2. The ICS results were reported as the frequencies (%) of NP-specific CD4+ or CD8+ T cells per respectively CD4+ T cells and CD8+ T cells. Final results below 0.0001% were set at 0.0001%.

## Haemagglutination inhibition assay (HAI)

The aim of the HAI assay was to detect antibodies, capable of inhibiting the agglutination between red blood cells (RBCs) and the viral hemagglutinin (HA). All serum aliquots were treated with receptor destroying enzyme (RDE) and incubated overnight at 37°C. Any remaining RDE in the sample was inactivated by heating (56°C, 1 h), before mixing with 12.5% RBC-phosphate buffer solution (PBS) solution (4°C, 1h). Treated sera were centrifuged and collected. A virus solution containing 4 HAU (hemagglutination units) in 25 µL was prepared and a back titration of the virus of interest was performed prior to start the assay. Treated sera were diluted two-fold from 1:1 to 1:2048 directly in the 96-multiwell plate, before adding the diluted virus. Finally, 50 μL of a 0.5% RBC-PBS solution was added to each well to measure agglutination inhibition. Results were reported as HAI titers represented by the reciprocal of the highest serum dilution in which agglutination was still completely inhibited.

The performance in terms of precision, linearity and range for the two evaluated strains A/ H1N1/Brisbane/02/2018 pdm09-like and A/H3N2/Kansas/14/2017 was established. Assay specificity was confirmed by mixing high HAI titer samples with low or negative serum samples. Assay intermediate precision and linearity were assessed by testing repeatedly high HAI titer anti-serum samples serially diluted (from net to 1:128 dilutions). The analytical range observed was 5120 to 10 (minimum and maximum values observed during linearity assay) for H1N1 and 1280 to 10 (minimum and maximum values observed during linearity assay) for H3N2. In those ranges, the assay met the criteria of linearity (coefficient of correlation ≥0.975 and slope between 0.8 and 1.2), intermediate precision (≤50%) and accuracy (% recovery between 50% and 200%).

# Supplementary Figures and Tables

## Supplementary Figures

**Supplementary Figure 1.** Flowchart of the OVX836-002 study (ILI = influenza-like illness; AE = adverse event; SAE = serious adverse event).


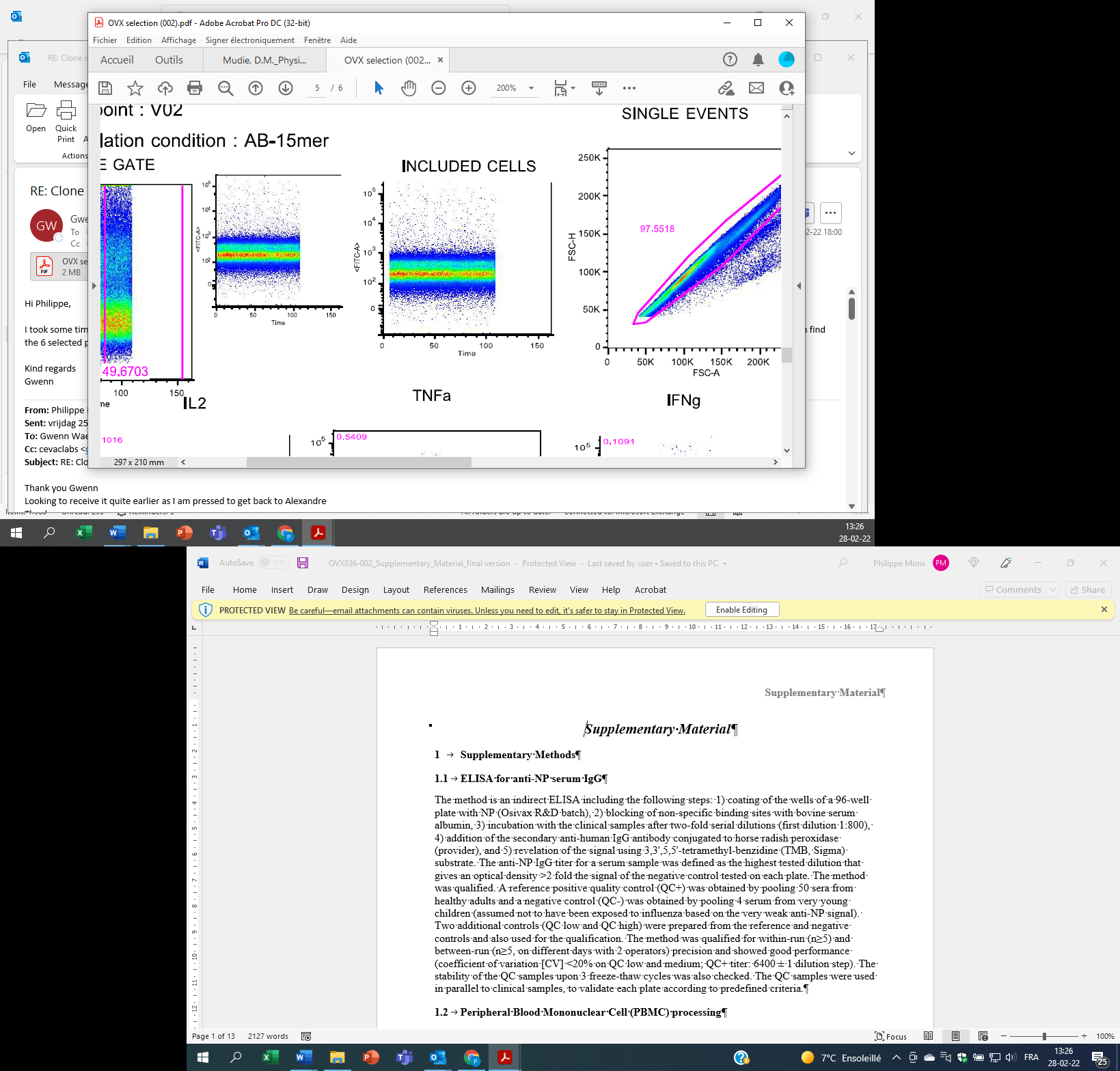

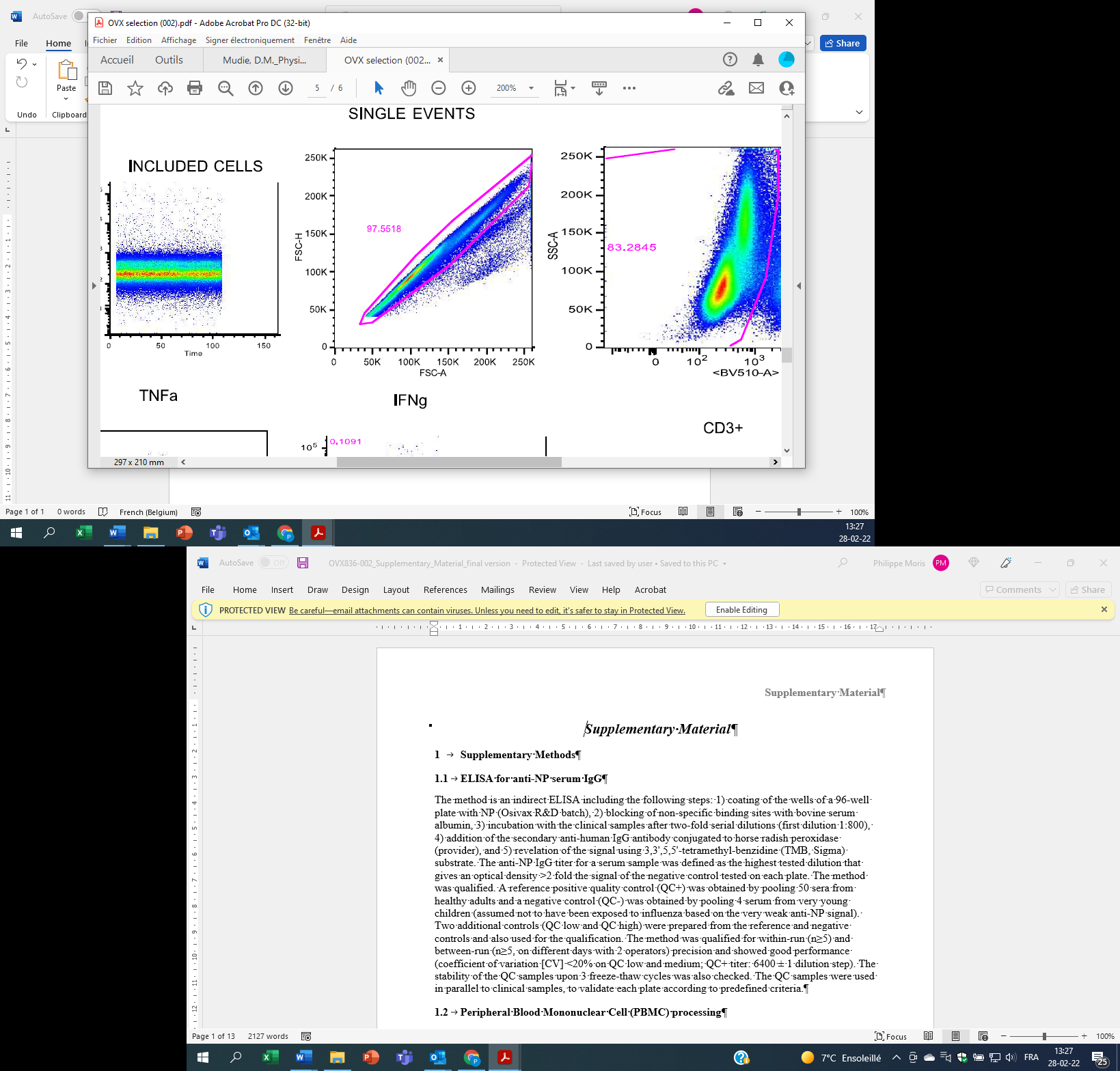

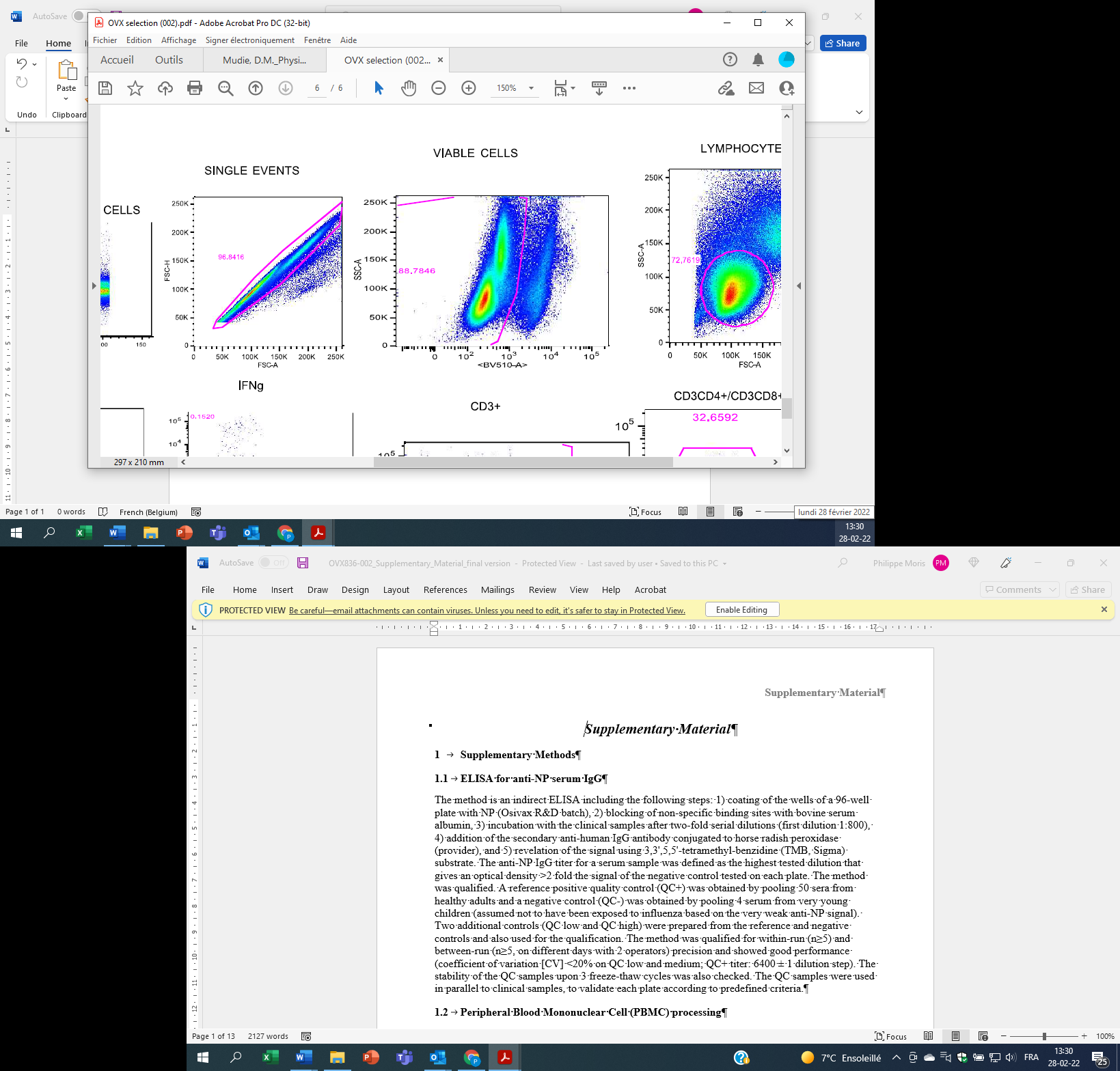


**A**

Live/Dead

SSC/A

FSC/H

FSC/A

Time

IFNg-Fitc


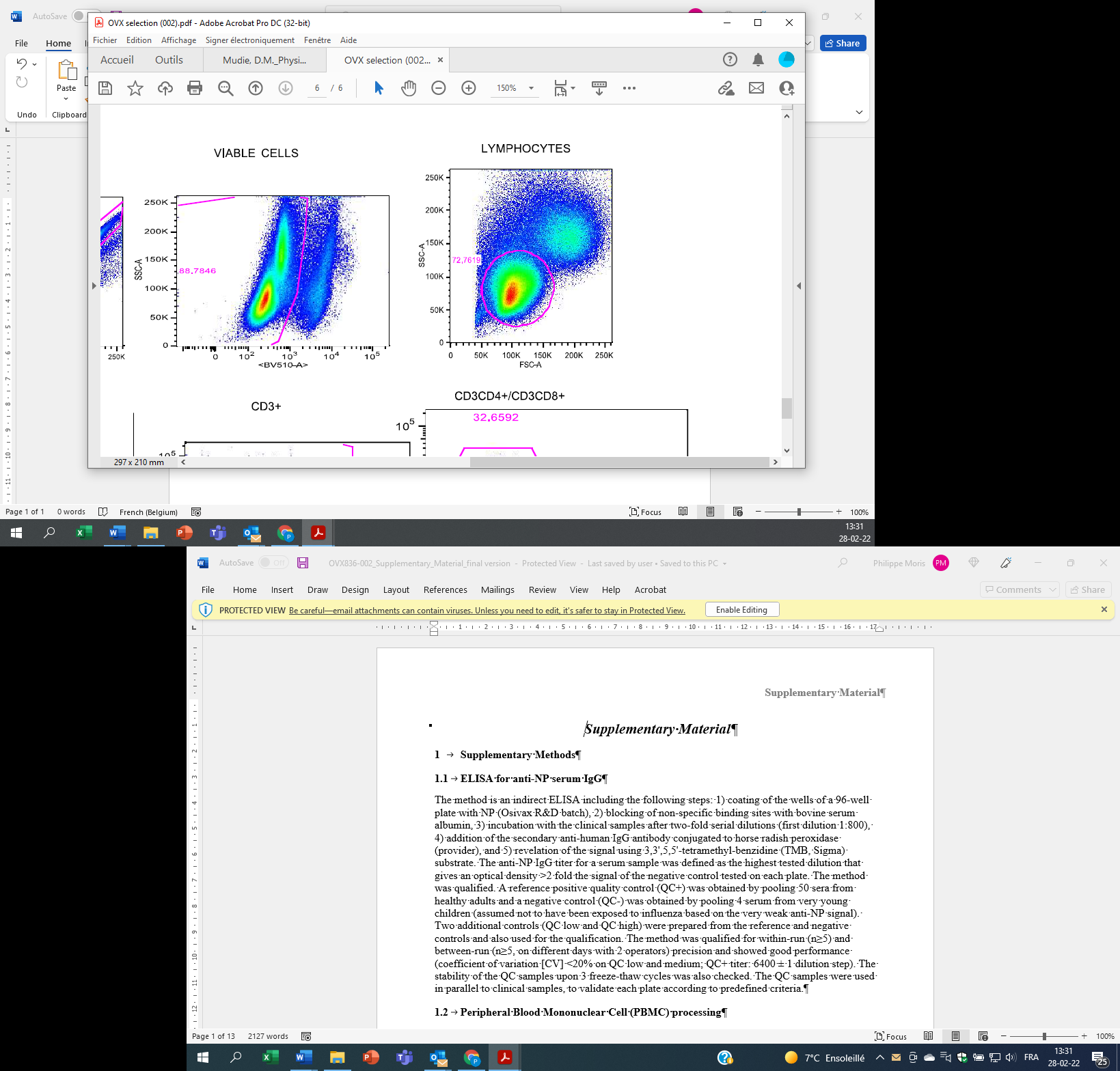

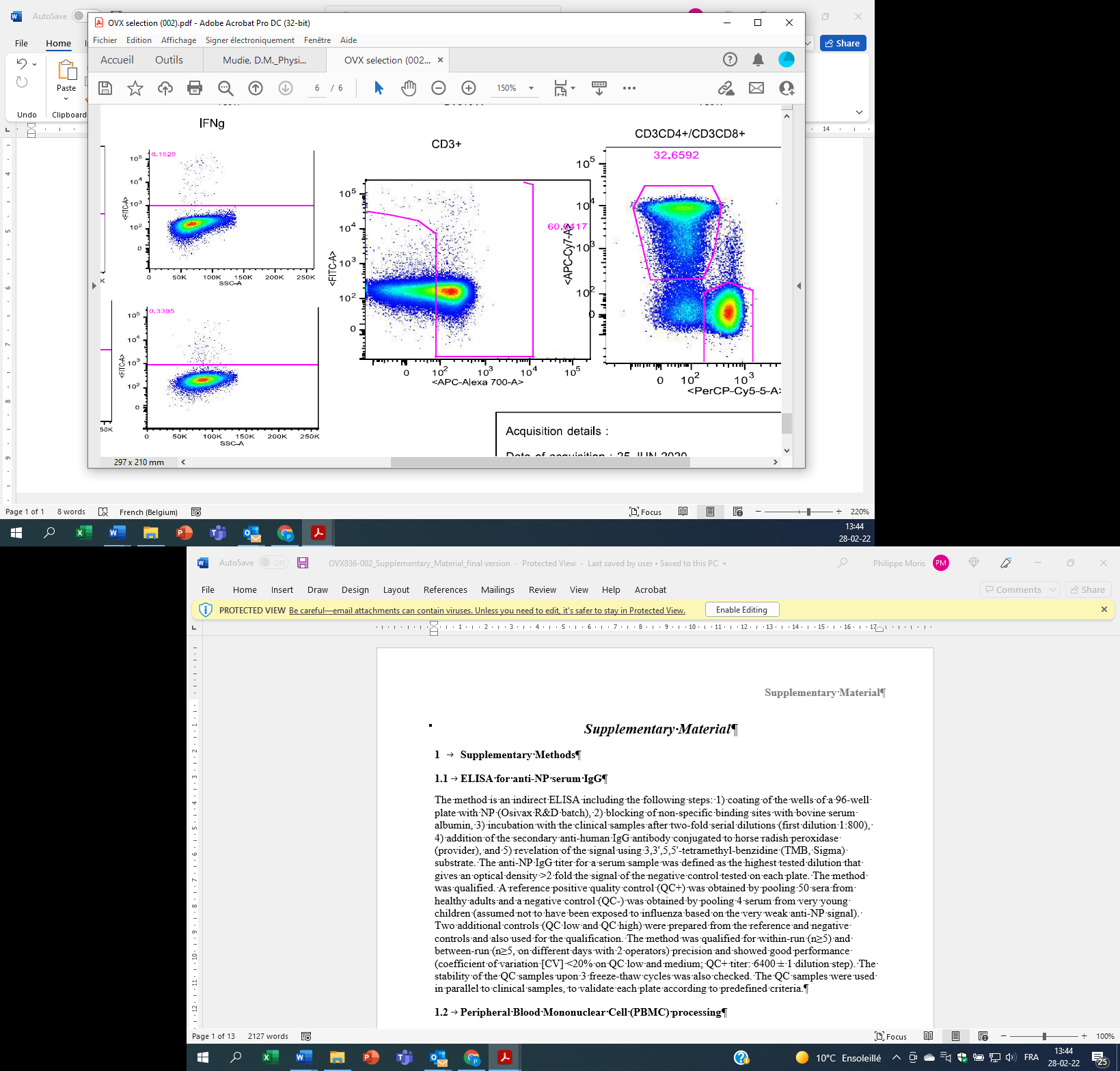

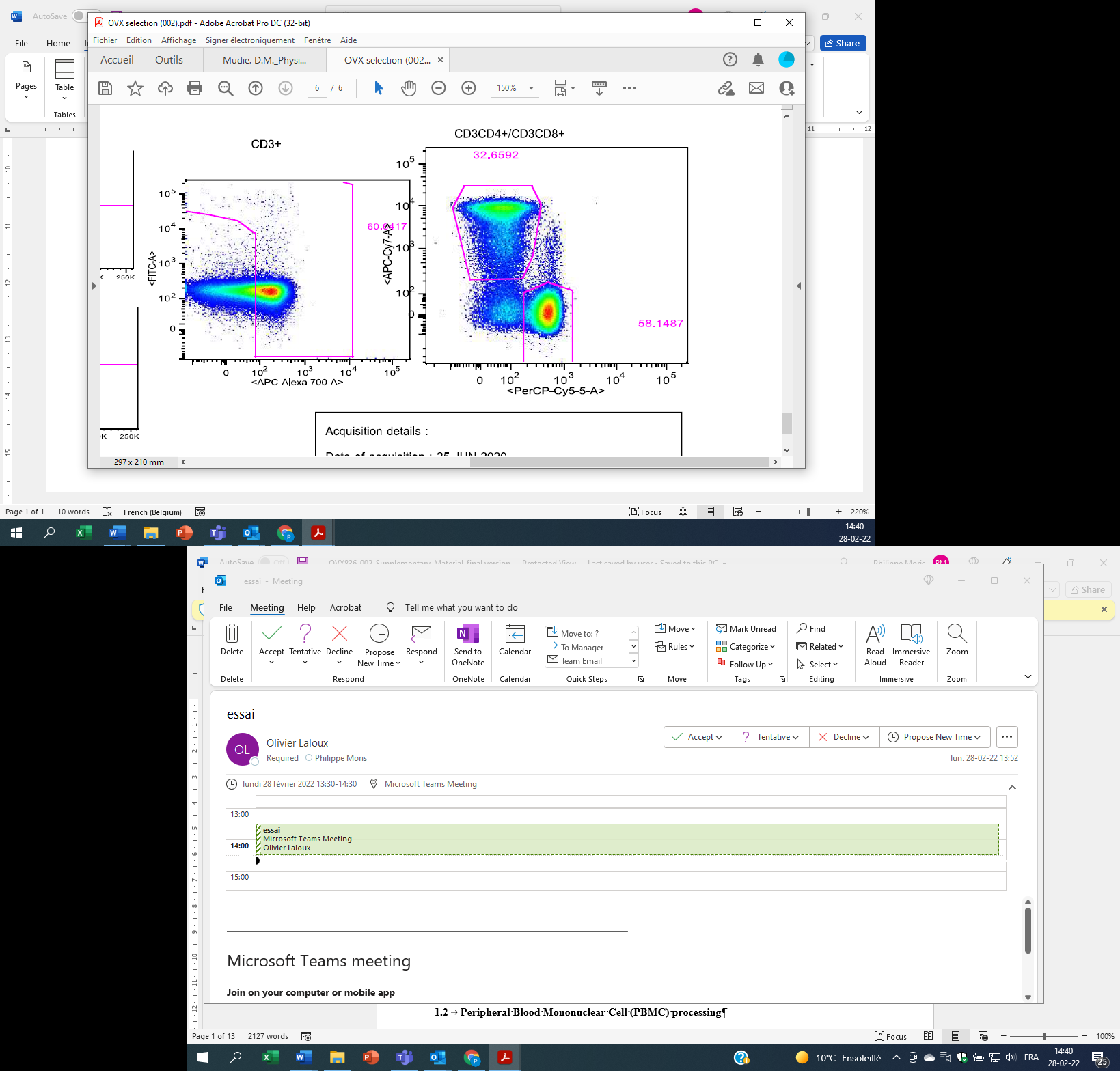


**B**

Analysis of Cytokine expression in CD3+CD4+

Analysis of Cytokine expression in CD3+CD8+

CD4

CD3

CD8

IFNg

FSC/A

SSC/A

CD3


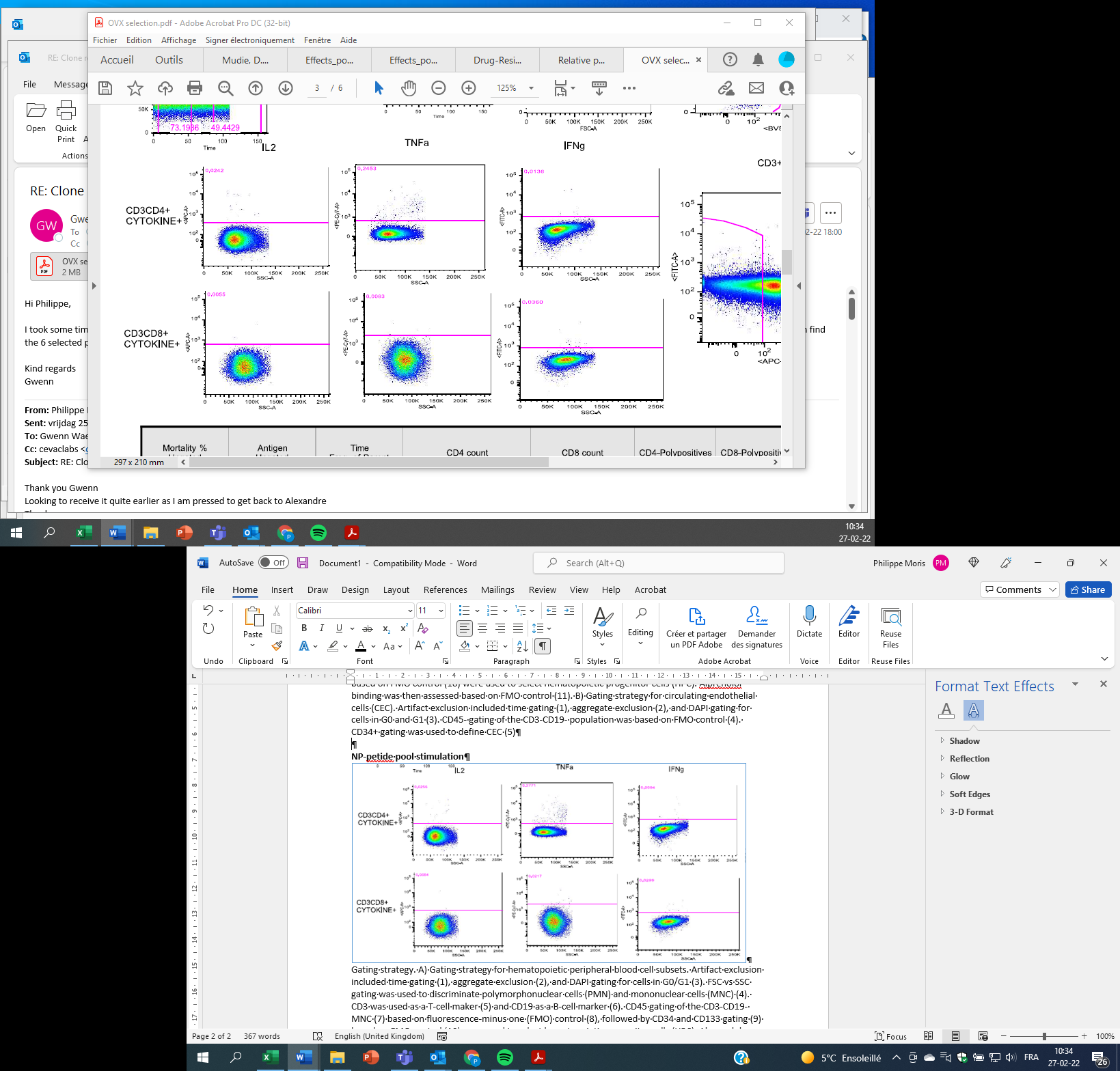


**C**

no stimulation IL2 TNFa IFNg


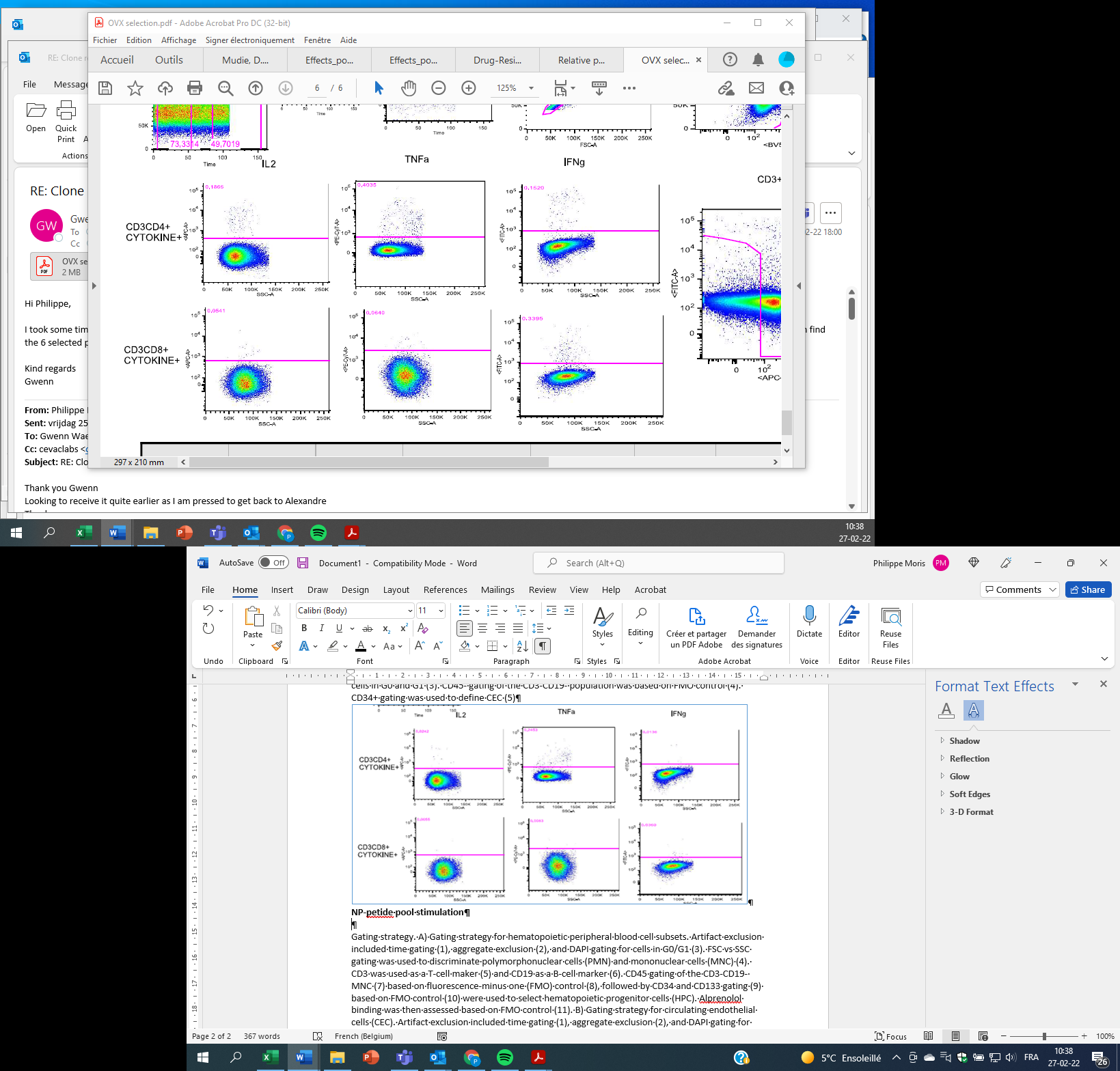


NP peptide stimulation IL2 TNFa IFNg

**Supplementary Figure 2.** Dot-plots in cytometry identifying specific cytokine expression in CD4+ and CD8+ events. (A) Gating strategy showing the sequential gating to identify CD3+CD4+ events and CD3+CD8+ events: Artifact exclusion included time gating (Time-IFNγ), aggregate exclusion (single events – FSC-/A-FSC/H), death cell exclusion (live-death-SSC/A), FSC/A-SSC/A gating to select mononuclear cells. CD3+events identified as CD3+ and dim CD3+/IFNγ+, leading to the gating of CD3+CD4+ and CD3+CD8+ events. (B) Illustrative dot plots showing expression of cytokines (IL-2, TNFα and IFNγ) in CD3+CD4+ and CD3+CD8+T cells upon in vitro culture with medium only (B) and upon in vitro culture with NP peptide pool (C).


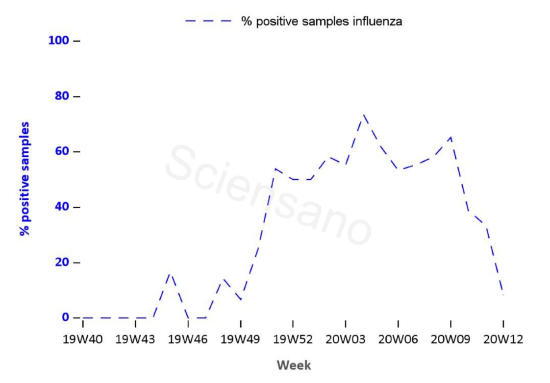


**Supplementary Figure 3.** Percentage of reverse transcriptase-polymerase chain reaction (RT-PCR)-confirmed influenza cases during the influenza season 2019-2020 in Belgium (Sciensano official publication; <https://www.sciensano.be/fr/file/virogppercflu1jpg>; accessed on 06 January 2022)

**Supplementary Figure 4.** CONSORT diagram of the OVX836-002 study presenting the number of subjects in the modified intention-to-treat and per protocol cohorts, distributed between the two age classes (18-49 years and 50-65 years) and the three treatment groups (Influvac Tetra^TM^, OVX836 90µg and OVX836 180µg) (PBMC = peripheral blood mononuclear cell; SAE = serious adverse event; RT-PCR = reverse transcriptase-polymerase chain reaction; COVID-19 = coronavirus disease-19).

**Supplementary Figure 5.** Evolution over time of the anti-nucleoprotein (NP)-specific immunoglobulin G (IgG) titers between baseline (Day 1 – pre-vaccination) and Day 180 (post-vaccination) in the three treatment groups (Influvac Tetra^TM^, OVX836 90µg and OVX836 180µg; per protocol cohort). Results are shown as geometric mean titers ± 95% confidence interval. Comparisons between the two OVX836 groups and the Influvac Tetra^TM^ (control) group at each timepoint: * p<0.05; ** p<0.01; **** p<0.0001. Panel A corresponds to all subjects, Panel B to subjects aged 18-49 years and Panel C to subjects aged 50-65 years.

**Supplementary Figure 6.** Evolution over time of the percentage of nucleoprotein (NP)-specific CD4+ T-cells positive at least for interferon-gamma (IFNγ) between baseline (Day 1 – pre-vaccination) and Day 180 (post-vaccination) in the three treatment groups (Influvac Tetra^TM^, OVX836 90µg and OVX836 180µg; per protocol cohort). Results are shown as means ± standard error. Comparisons between the two OVX836 groups and the Influvac Tetra^TM^ (control) group at each timepoint: * p<0.05; ** p<0.01; **** p<0.0001. Panel A corresponds to all subjects, Panel B to subjects aged 18-49 years and Panel C to subjects aged 50-65 years.

**Supplementary Figure 7.** A. Percentage of nucleoprotein (NP)-specific CD8+ T-cells positive at least for interferon-gamma (IFNγ) at baseline (Day 1 – pre-vaccination) in the three treatment groups (Influvac Tetra^TM^, OVX836 90µg and OVX836 180µg) at the different dates of subject inclusion into the study (before and during the influenza season). B. Median of the percentage of NP-specific CD8+ T-cells positive for IFNγ at baseline (D1 = Day 1 – pre-vaccination) and on Day 8 (D8 post-vaccination) in the three treatment groups (Influvac Tetra^TM^, OVX836 90µg and OVX836 180µg) and in the subjects (modified intention-to-treat cohort) belonging to the lowest quartile of the CD8+ response at baseline. Paired comparisons between timepoints; ** p<0.01.

**Supplementary Figure 8.** Kaplan-Meier survival analysis of the cumulative risk of influenza-like illness (ILI) as a function of time between vaccination and first ILI episode (day) during the influenza season (defined as the period between 02 December 2019 and 09 March 2020) in the three treatment groups (Influvac Tetra^TM^, OVX836 90µg and OVX836 180µg; modified intention-to-treat cohort corresponding to the safety cohort).

## Supplementary Tables

**Supplementary Table 1. Demographics and baseline subject characteristics of the subjects**

| **All subjects** | | **OVX836**  **90 µg (N=100)** | **OVX836**  **180 µg (N=101)** | **Influvac**  **Tetra (N=99)** | **All (N=300)** |
| --- | --- | --- | --- | --- | --- |
| Age (years) | Mean (SD) | 39.8 (13.4) | 40.1 (13.8) | 40.4 (13.5) | 40.1 (13.5) |
| Sex | Female, n (%) | 73 (73.0) | 67 (66.3) | 61 (61.6) | 201 (67.0) |
| Body Mass Index (kg/m^2^) | Mean (SD) | 24.9 (3.7) | 24.0 (3.3) | 24.7 (3.5) | 24.5 (3.5) |
| Smoking status | Smoker, n (%) | 12 (12.0) | 10 (9.9) | 7 (7.1) | 29 (9.7) |
|  | Ex-smoker, n (%) | 18 (18.0) | 14 (13.9) | 15 (15.2) | 47 (15.7) |
|  | Nonsmoker, n (%) | 70 (70.0) | 77 (76.2) | 77 (77.8) | 224 (74.7) |
| **Subjects aged 18-49 years** | | **N=69** | **N=70** | **N=69** | **N=208** |
| Age (years) | Mean (SD) | 32.2 (8.2) | 32.5 (8.8) | 33.0 (8.2) | 32.6 (8.4) |
| Sex | Female, n (%) | 52 (75.4) | 46 (65.7) | 39 (56.5) | 137 (65.9) |
| Body Mass Index (kg/m^2^) | Mean (SD) | 23.7 (3.0) | 23.7 (3.0) | 24.2 (3.3) | 23.9 (3.1) |
| Smoking status | Smoker, n (%) | 8 (11.6) | 6 (8.6) | 6 (8.7) | 20 (9.6) |
|  | Ex-smoker, n (%) | 9 (13.0) | 7 (10.0) | 6 (8.7) | 22 (10.6) |
|  | Nonsmoker, n (%) | 52 (75.4) | 57 (81.4) | 57 (82.6) | 166 (79.8) |
| **Subjects aged 50-65 years** | | **N=31** | **N=31** | **N=30** | **N=92** |
| Age (years) | Mean (SD) | 56.5 (4.0) | 57.3 (4.3) | 57.5 (4.4) | 57.1 (4.2) |
| Sex | Female, n (%) | 21 (67.7) | 21 (67.7) | 22 (73.3) | 64 (69.6) |
| Body Mass Index (kg/m^2^) | Mean (SD) | 27.5 (3.8) | 25.0 (3.8) | 25.8 (3.7) | 26.1 (3.9) |
| Smoking status | Smoker, n (%) | 4 (12.9) | 4 (12.9) | 1 (3.3) | 9 (9.8) |
|  | Ex-smoker, n (%) | 9 (29.0) | 7 (22.6) | 9 (30.0) | 25 (27.2) |
|  | Nonsmoker, n (%) | 18 (58.1) | 20 (64.5) | 20 (66.7) | 58 (63.0) |

SD = standard deviation

**Supplementary Table 2. Most frequent adverse events reported by the subjects in the three treatment groups during the 28-day period after vaccination, classified by MedDRA System Organ Class and Preferred Term**

| **Primary System Organ Class** | **Dictionary-Derived Term** | **OVX836 90µg (N=100)** | **OVX836 180µg (N=101)** | **INFLUVAC TETRA (N=99)** |
| --- | --- | --- | --- | --- |
| Blood and Lymphatic System Disorders | Lymphadenopathy | 0 (0.0%) [0.0, 3.6] | 2 (2.0%) [0.2, 7.0] | 5 (5.1%) [1.7, 11.4] |
| General Disorders and Administration Site Conditions | Influenza Like Illness | 12 (12.0%) [6.4, 20.0] | 12 (11.9%) [6.3, 19.8] | 8 (8.1%) [3.6, 15.3] |
| Infections and Infestations | Upper Respiratory Tract Infection | 7 (7.0%) [2.9, 13.9] | 7 (6.9%) [2.8, 13.8] | 3 (3.0%) [0.6, 8.6] |
| Nervous System Disorders | Headache | 7 (7.0%) [2.9, 13.9] | 9 (8.9%) [4.2, 16.2] | 6 (6.1%) [2.3, 12.7] |
| Respiratory, Thoracic and Mediastinal Disorders | Oropharyngeal Pain | 5 (5.0%) [1.6, 11.3] | 2 (2.0%) [0.2, 7.0] | 3 (3.0%) [0.6, 8.6] |

N = Number of subjects. Results are given as (n) number and (%) percentage of subjects reporting the concerned adverse event; [lower limit, upper limit] of the exact 95% confidence interval

**Supplementary Table 3. Influence of the baseline (pre-vaccination) immunological parameters on the occurrence of influenza-like illness during the 13-day period following vaccination in all subjects, irrespective of the treatment group**

|  | **Occurrence of ILI** | | | |  |
| --- | --- | --- | --- | --- | --- |
|  | **No** | | **Yes** | |  |
|  | **N** | **Mean** ± SD | **N** | **Mean** ± SD | **P value** |
| Anti-NP IgG | 276 | 7632 ± 8145 | 23 | 6539 ± 5035 | >0.05 |
| IFNγ-spot forming cells/10^6^ PBMC | 276 | 150 ± 184 | 23 | 69 ± 49 | <0.0001 |
| % of CD8+ T-cells producing at least IFNγ | 274 | 0.197 ± 0.232 | 23 | 0.126 ± 0.112 | >0.05 |
| % of CD4+ T-cells producing at least IFNγ | 276 | 0.034 ± 0.034 | 23 | 0.022 ± 0.011 | <0.0001 |

N = Number of subjects; Results are given as mean ± standard deviation; P value from Independent Student ‘s t tests.
